# Infection of *Drosophila suzukii* with the obligate insect-pathogenic fungus *Entomophthora muscae*

**DOI:** 10.1007/s10340-017-0915-3

**Published:** 2017-09-09

**Authors:** Paul G. Becher, Rasmus E. Jensen, Myrsini E. Natsopoulou, Vasiliki Verschut, Henrik H. De Fine Licht

**Affiliations:** 1 Chemical Ecology Unit, Department of Plant Protection Biology, Swedish University of Agricultural Sciences, Box 102, 23053 Alnarp, Sweden; 2 Section for Organismal Biology, Department of Plant and Environmental Sciences, University of Copenhagen, Thorvaldsensvej 40, 1871 Frederiksberg, Denmark; 3 Section for Entomology and Plant Pathology, Department of Agroecology, Aarhus University, Forsøgsvej 1, 4200 Slagelse, Denmark

**Keywords:** Entomopathogen, Fly, Fungal pathogen, Insect pest, Spotted wing drosophila

## Abstract

Physiological constraints restrict specialist pathogens from infecting new hosts. From an applied perspective, a narrow host range makes specialist pathogens interesting for targeting specific pest insects since they have minimal direct effects on non-target species. Entomopathogenic fungi of the genus *Entomophthora* are dipteran-specific but have not been investigated for their ability to infect the spotted wing drosophila (SWD; *Drosophila suzukii*) a fruit-damaging pest invasive to Europe and America. Our main goal was to study whether SWD is in the physiological host range of the entomophthoralean species *E. muscae*. We investigated pathogenicity and virulence of *E. muscae* towards its main natural host, the housefly *Musca domestica*, and towards SWD. We found that *E. muscae* readily infected and significantly reduced survival of SWD by 27.3% with the majority of flies dying 4–8 days post-exposure. In comparison with SWD, infection of the natural host *M. domestica* resulted in an even higher mortality of 62.9% and larger conidial spores of *E. muscae*, reflecting the physiological constraints of the pathogen in the atypical host. We demonstrated that pathogens of the *E. muscae* species complex that typically have a narrow natural host range of one or few dipteran species are able to infect SWD, and we described a new method for in vivo transmission and infection of an entomophthoralean fungus to SWD.

## Key message

- Dipteran-specific pathogens are potential agents to control the fruit-damaging *Drosophila suzukii*.
- This is the first report of the obligate insect-pathogenic fungus *Entomophthora muscae* being able to infect and kill male and female *D. suzukii*.
- An even higher virulence towards the natural host *Musca domestica* and differences in spore morphology may reflect physiological constraints of the tested *E. muscae* isolate when infecting *D. suzukii*.
- *Entomophthora* species like the dipteran-specific *E. muscae* have a potential for biological control of *D. suzukii*.

## Introduction

The Asian spotted wing drosophila (SWD; *Drosophila suzukii*) is an invasive and serious economic pest in fruit and berry. Since growers became aware of a SWD invasion in Southern Europe and the Eastern USA in 2008, the fly expanded its geographic range dramatically within Europe and the Americas (Asplen et al. 2015).

During geographic expansion, invasive insects come into contact with previously un-encountered pathogens that try to exploit the new species as a host. However, virulent pathogens, parasites and predators can be sparse or unable to regulate populations of pest species at early states of invasion as exemplified by the devastating dispersal of SWD. Pathogens that are able to develop diseases in a new invasive pest consequently are of potential value to suppress population densities and thus the impact of the pest. Active suppression of pest populations can be approached through different strategies of biological control ranging from measures that protect or enhance specific antagonists in the environment, to the intentional release of control agents (Eilenberg et al. 2001). Antagonists such as entomopathogenic fungi are generally accepted as a safer alternative to chemical insecticides, although direct and indirect ecological effects on non-target organisms are common and need to be considered in risk assessment of any control measure (Flexner et al. 1986; Cory and Myers 2000; Goettel and Hajek 2000; Shah and Pell 2003). Negative effects on beneficial and other non-target arthropods were for example shown for entomopathogenic fungi from the genera *Metarhizium* and *Beauveria* (Vestergaard et al. 2003).

Entomopathogenic fungi are common in nature, have significant impact on insect populations and are successfully applied as biological control agents (Hajek and St. Leger 1994; Goettel et al. 2005; Wang and Wang 2017). Insect-pathogenic fungi differ in the natural range of host species they infect and are often designated as generalists or specialists (Boomsma et al. 2014). The wide range of host species used by generalist insect-pathogenic fungi, such as many species within the genera *Metarhizium* and *Beauveria*, imply that these fungi are likely to make ‘host-shifts’ onto newly encountered hosts. Consequently, hypocrealean fungi such as *M. robertsii* and *B. bassiana* are commonly applied for insect control (Ferron 1981; Meyling and Eilenberg 2007), and commercially available fungal biological control agents based on generalist entomopathogenic fungi have been studied for control of SWD (Woltz et al. 2015; Cossentine et al. 2016; Cuthbertson and Audsley 2016). Collectively these studies show that SWD can be infected and killed by several different insect-pathogenic fungi. In particular, a recent study showed high insecticidal activity of *Metarhizium brunneum* when applied in specifically designed lures to infect and kill SWD (Yousef et al. 2017).

Because of limitations in time-to-kill and difficulty with application of infective conidia, insect-pathogenic fungi are generally used in combination with other measures as part of an integrated pest management (IPM) strategy (Haye et al. 2016; Shah and Pell 2003). To avoid disruption of current IPM strategies in fruit and berry control that for example may involve non-target effects on predators and parasitoids, pest-specific pathogens would be desirable but have so far not been explored for biological control of SWD (Cuthbertson and Audsley 2016; Hamby and Becher 2016; Yousef et al. 2017). Diptera-infecting entomophthoralean fungi (Jensen et al. 2006; Vega et al. 2012) are known to cause natural epizootics, killing large numbers of insects and can decimate pest populations (Roberts and Humber 1981). Entomophthoralean fungi in the *Entomophthora muscae* species complex are morphologically distinguishable based on the number of nuclei in conidia and include *E. muscae, E. schizophorae* and *E. syrphi* (Keller et al. 1999; Jensen et al. 2006, 2009). Each species is, in contrast to generalist hypocrealean fungi such as *M. robertsii* and *B. bassiana*, considered to have narrow natural host ranges. Within *Entomophthora* species, individual populations are genetically distinct as for example isolates of *E. muscae* from cabbage fly (*Delia radicum*) are genetically distinct from *E. muscae* isolates from house flies (*Musca domestica*) (De Fine Licht et al. 2017; Jensen et al. 2001). Despite high specificity, isolates of several *Entomophthora* species are capable of infecting other species of diptera than the natural host (Jensen et al. 2006). *Entomophthora schizophorae* (isolate originally described as *E. muscae*) from housefly (*M. domestica*) is for example able to infect the common fruit fly (*D. melanogaster*) at low prevalence (Steinkraus and Kramer 1987; Keller 2007).

Here, our main goal was to investigate whether SWD is in the physiological host range of the entomophthoralean fungus, *Entomophthora muscae s. str.* (here after called *E. muscae*), which is an important natural enemy of the common housefly, *Musca domestica* (Kalsbeek et al. 2001). *E. muscae* is an obligate insect-pathogen that grows as protoplasts inside the fly host. After typically ca. 6–7 days, *E. muscae* takes over the behaviour of infected hosts and forces them to seek out elevated positions. The host is eventually killed in a characteristic posture with wings spread away from the abdomen, while *E. muscae* grows out through the intersegmental membranes in the abdomen where it releases infective conidia (Gryganskyi et al. 2017; Hansen and De Fine Licht 2017). *Entomophthora muscae* causes natural epizootics in housefly populations (Kalsbeek et al. 2001), and here we explored the infectivity of *E. muscae* towards SWD. In the laboratory, we tested for infection of SWD with *E. muscae* by direct exposure to sporulating housefly cadavers and documented pathogenicity, virulence and conidia morphology of *E. muscae*-infected SWD.

## Materials and methods

### Isolates and flies

House flies (*M. domestica*, strain: 772a) were provided as pupae from the Department of Agroecology, Aarhus University, Denmark. Flies of SWD (*D. suzukii*) originated from a laboratory strain maintained at SLU, Alnarp on a cornmeal diet (Revadi et al. 2015). *Entomophthora muscae* isolate hhdfl130914-01, that was originally obtained from a dead infected *M. domestica* collected in a cow byre near Slangerup, Sealand, Denmark (Hansen and De Fine Licht 2017), and is deposited in the insect-pathogenic fungal culture collection at Department of Plant and Environmental Sciences, University of Copenhagen (acc. no. KVL-14-115). The *E. muscae* isolate was maintained in vivo by continuous infections in house flies as previously described (De Fine Licht et al. 2017). Briefly, house flies were kept in groups of 20–40 flies of mixed sex in containers with diameter: 7.5 cm, height: 8 cm. Containers were closed with insect net and administered with water and dry yeast and sugar mixed 1:6 and kept at 21 ± 1 °C. For infection, three fresh (dead <12 h) *E. muscae*-sporulating fly cadavers actively discharging conidia were placed at the top of the container for 24 h at ca. 100% humidity. After 7 days’ post-exposure, dead, infected and sporulating fly cadavers were removed from containers and used to infect new healthy flies.

### Experimental set-up

Adult 3-day-old SWD flies were exposed to fresh *M. domestica* cadavers infected with *E. muscae.* For infecting SWD, two dead sporulating housefly cadavers were fixed with Vaseline underneath the cotton lid inside a *Drosophila* food vial for 24 h. Control treatments consisted of the exact same set-up, except two uninfected housefly cadavers were fixed at the lid with Vaseline. Each *Drosophila* food vial contained 12–27 unmated male or female SWD flies, with eight replicate vials per treatment. Two strips of filter paper were added within each vial to facilitate climbing of infected flies. Vials were kept at room temperature (23 °C ± 2), with a photoperiod of 12:12 (L:D). Number of dead SWD flies and inspection of cadavers for presence of external fungal growth and general observations were recorded daily for 10 days. To obtain comparable infectivity measurements for *E. muscae* infections in house flies, vials of diameter: 7.5 cm, height: 8 cm with 28–46 house flies were similarly exposed for 24 h to two sporulating *E. muscae-*infected housefly cadavers. Number of dead house flies and inspection of cadavers for presence of external fungal growth were recorded daily for 10 days as described for SWD.

### Conidia exposure dosage and conidia morphology

The exposure dosage of *E. muscae* conidia during the 24-h infection scheme was calculated based on eleven sporulating housefly cadavers placed individually over a 1-ml solution containing 1% Triton-X and 0.2% maleic acid to prevent germination of discharged conidia (Hajek et al. 2012). Following 24-h exposure, conidia were counted using a hemocytometer placed under a microscope. To examine conidia morphology in the two different hosts, *E. muscae-*infected cadavers of house flies and SWD were placed on microscope slides at high humidity to induce discharge of conidia onto the microscope slides. Microscope slides with conidia were stained with aceto-orcein and examined with a microscope at 100× magnification. Length, width and number of nuclei within individual conidia from housefly and SWD cadavers were measured.

### Statistical analyses

Generalized linear models (GLMs) with a binomial distribution were used to analyse the effect of the fungus on the proportion of overall mortality of adult flies. The models included treatment (*E. muscae* application vs. control), species (*D. suzukii* vs. *M. domestica*) and sex of the flies as explanatory variables. Model selection was performed using likelihood ratio tests based on X2 and Akaike’s information criterion in a stepwise backward selection process from full models testing main effects and two-way interactions between the explanatory variables. Pairwise comparisons were performed using Tukey’s HSD post hoc test, with a Bonferroni correction.

In order to test the effect of *E. muscae* on the survival of *D. suzukii* and *M. domestica* in a time dependent manner the Cox proportional hazards (PH) regression model (Cox 1972) was used. Due to very low percentage of mortality in the control groups of both fly species, which resulted in high levels of censored data, these groups were excluded and survival was analysed as a function of species and sex of the flies. Differences in number of nuclei between *E. muscae* conidia from house flies and fruit flies were analysed with a Wilcoxon rank-sum test, whereas conidia differences in length, width and aspect ratio were analysed using Student’s *t* tests after log-transforming data. All analyses were carried out in R (v. 3.3.0; R Core Team 2013) using the packages car, multcomp and survival.

## Results

### *Entomophthora muscae* infections of *D. suzukii*

**Fig. 1:**
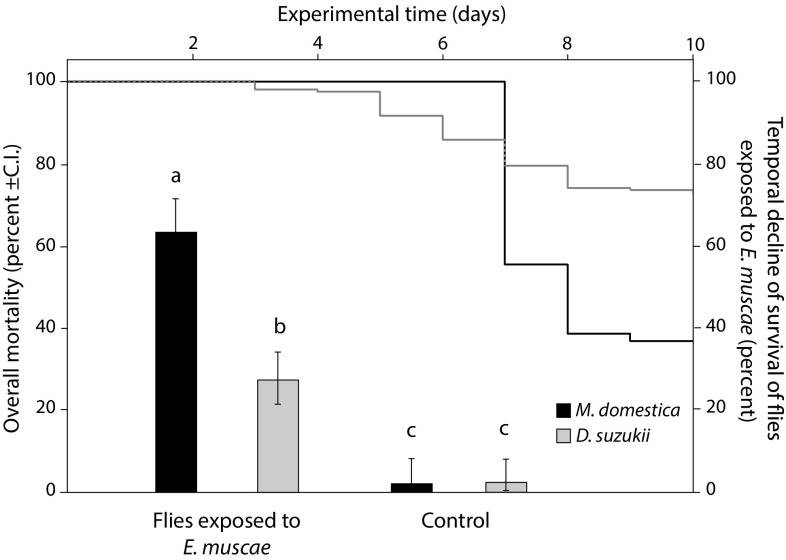
Percentage of overall mortality over 10 days (bars ±SE) and temporal decline of survival (lines) in *M. domestica* (black) and *D. suzukii* (grey) following 24 h exposure to housefly cadavers with *E. muscae* conidiospores and uninfected control cadavers. Exposure to *E. muscae* conidia had a significant effect on both *M. domestica* and *D. suzukii* survival. The letters above each *bar* denote significantly different overall mortality percentage

**Fig. 2:**
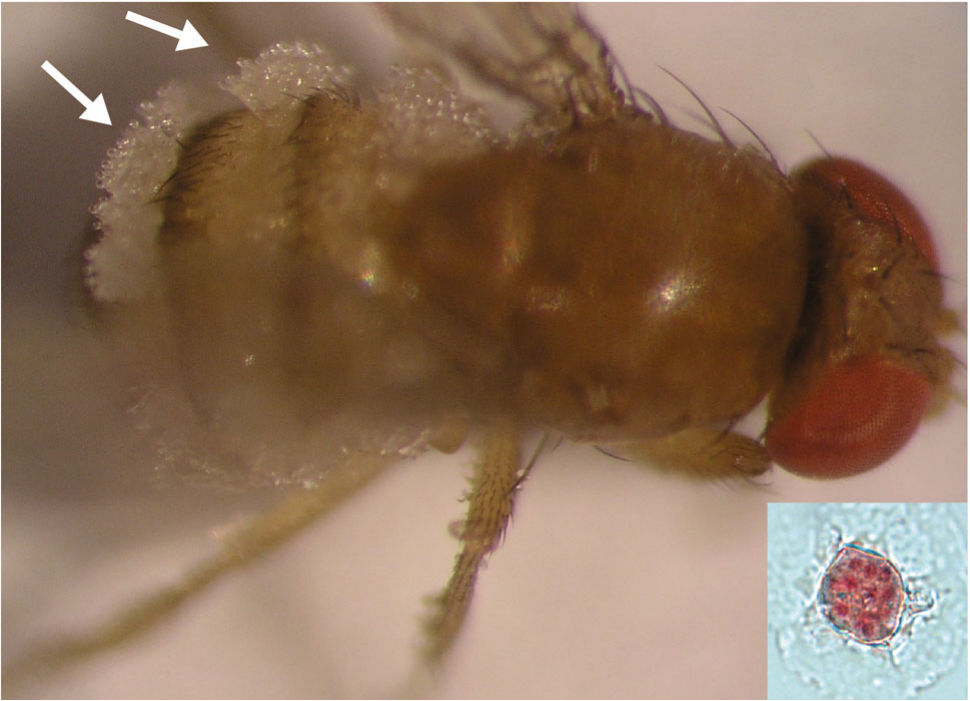
Abdomen of dead *D. suzukii* with conidiophores of *E. muscae* growing out between the tergites and sclerites of the abdomen. Insert shows an *E. muscae* spore from *D. suzukii* with the characteristic *Entomophthoralean* oval shape of a rounded base with a pointed apex. Several nuclei can be seen inside

A significant effect of treatment (χ^2^ = 130.027, df = 1, *P* < 0.001) and species (χ^2^ = 37.696, df = 1, *P* < 0.001) was observed on the overall mortality of flies, while sex did not affect the response variable (χ^2^ = 3.316, df = 1, *P* = 0.068). All pairwise comparisons revealed significant differences (*P* < 0.001) except from the comparison between the control groups of the two species (*z* = 0.263, *P* = 0.993) (Fig. 1). From a total of 205 SWD flies exposed to infected *M. domestica* cadavers, 56 died within 10 days, from which, 53.6% (C.I. 39.7–67.0%) developed visible external mycelium (Fig. 2). Moreover, in several SWD infection induced characteristic behavioural symptoms with flies climbing elevated positions where they died with wings raised above the body confirming the involvement of the fungus. However, the timing of this behavioural manipulation and death was less synchronized in SWD than house flies, with SWD starting to die earlier than *M. domestica* but with mortality distributed over more days (Fig. 1). In the respective group of *M. domestica*, from a total of 124 flies exposed to sporulating *M. domestica* cadavers, 78 died, all of which developed visible conidia. Analysis with the Cox PH regression model revealed a significant effect of species on the survival of flies exposed to infected *M. domestica* cadavers (χ^2^ = 32.5794, df = 1, *P* < 0.001), while sex of the flies was marginally non-significant (χ^2^ = 3.5836, df = 1, *P* = 0.0584).

### Conidia exposure dosage and morphology

In our experimental set-up, a single *E. muscae-*sporulating housefly cadaver produces 2.25×10^6^ ± 3.46×10^5^ conidia (mean ± SE, *N* = 11) during the first 24 h. The SWD and *M. domestica* vials and containers were therefore exposed to a minimum dosage of 4.50×10^6^ conidia over the 24 h. There was no difference in number of nuclei between conidia from *E. muscae* when infecting the natural host *M. domestica* and the experimental host SWD (*W* = 149.5, *p* = 0.249, Table 1). In contrast, mean length and width of the conidia were significantly smaller on SWD than on *M. domestica* (*t* = 2.66, df = 37.4, *p* = 0.012; *t* = 5.08, df = 38.0, *p* < 0.001, respectively). The shape of conidia measured as the aspect ratio between length and width of the conidia was similarly significantly different between conidia from *E. muscae* when infecting the natural host *M. domestica* and the experimental host SWD (*t* = 4.37, df = 27.9, *p* < 0.001), with a wider range of aspect-ratios present in conidia from SWD (Table 1).

**Table 1:** Spore morphology of *E. muscae* on the natural host (*M. domestica*) and the experimental host (*D. suzukii*)

Host species	Mean no. of nuclei	Mean length (µm)	Mean width (µm)	Mean aspect ratio
*M. domestica*	12.6 ± 0.3 (11–16)	30.4 ± 0.5 (26.2–34.5)	25.9 ± 0.4 (23.2–29.6)	1.17 ± 0.01 (1.11–1.23)
*D. suzukii*	13.3 ± 0.5^a^ (11–16)	28.5 ± 0.6 (25.2–36.8)	22.8 ± 0.4 (19.7–27.6)	1.25 ± 0.02 (1.15–1.39)
*t* test (*t*)/Wilcoxon (*W*)	*W* = 149.5 *p* = 0.249	*t* = 2.66, df = 37.4 *p* = 0.012	*t* = 5.08, df = 38.0 *p* < 0.001	*t* = 4.37, df = 27.9^b^ *p* < 0.001

The mean (*n* = 20) with standard error of the mean and the range in brackets are given

^a^ Nuclei could only be counted in 12 *E. muscae* spores from *D. suzukii*

^b^ Aspect ratios were log-transformed to normalize data before performing Student’s *t* test

## Discussion

The spotted wing drosophila is a most prominent example of insect species that currently invade new geographic regions where they become pests through fast increase in distribution and abundance. Management of invasive pests is a challenge that requires understanding of physiological and ecological mechanisms underlying their dispersal and invasion, and the development of tools to control their impact in natural and agricultural systems (Cini et al. 2014; Hamby et al. 2016).

The release from natural enemies like pathogens and predators is regarded as an important factor contributing to the establishment of invasive species in new habitats (Keane 2002; Comont et al. 2014). Pathogens, as one category of natural antagonists, consequently are applied to counteract enemy release. Entomopathogenic fungi in the orders Hypocreales and Entomophthorales are the most commonly used pathogens for biocontrol of insect pests. Biological control strategies using entomopathogenic fungi range from the approach of protecting and enhancing natural enemies already present in the environment to the intentional release of exotic control agents (Eilenberg et al. 2001; Pell et al. 2010). Sustainable control of SWD that do not disrupt currently employed IPM strategies requires the development of new strategies. Undoubtedly, our ecosystems host many pathogenic fungi of unknown value for control of pest populations (Pell et al. 2010), and here we wanted to know if SWD is in the host range of the entomophthoralean fungus *E. muscae* as a basis for the potential application of entomophthoralean entomopathogens as biocontrol agents. In the present study, we therefore explored the physiological host range by exposing SWD to *E. muscae* from house flies and demonstrated that *E. muscae* is able to infect, behaviourally manipulate and sporulate in SWD.

Higher infectivity and a larger spore size in house flies than in SWD likely illustrates special adaptations of *E. muscae* to the main natural host *M. domestica*. Nevertheless, infected SWD, similar to house flies, showed climbing and posturing of the abdomen with conidiospores growing out between the tergites and sclerites, to get actively discharged. In the natural housefly host, *E. muscae* disease development is characterized by initial exponential growth (Hansen and De Fine Licht 2017), immune avoidance by proliferating as protoplasts without cell walls (Latge et al. 1988) and behavioural manipulation of hosts to enhance transmission at the final stages of infection (Roy et al. 2006; Gryganskyi et al. 2017). Although less synchronized in time-to-kill, the disease ontogeny and complex behavioural manipulation of *E. muscae* in SWD is similar to infections in housefly. The near-natural *E. muscae* infection of SWD is consistent with previous work that also documented the potential host range of *E. muscae* being broader than the known natural host range (Jensen et al. 2006) similar as for *E. schizophorae* (isolate originally designated *E. muscae*) that was shown to be infectious for another *Drosophila* species, *D. melanogaster* (Steinkraus and Kramer 1987).

Infection of hosts outside the recorded natural host range is known for other infectious pathogens and likely reflects optimized laboratory conditions for pathogen transmission rarely experienced in nature. Under natural conditions, examples of non-host infections are often pathogen spill-over events without prolonged ecological persistence in the new host population (Poulin et al. 2011). Remarkably, specific *E. muscae* isolates have been described to cause high natural infection levels and epizootics in other dipteran pests like the carrot fly *Psila rosae* or the onion fly *Delia antiqua* (Carruthers et al. 1985; Eilenberg and Philipsen 1988). Interestingly, carrot flies caught in the hedgerow showed higher infection levels than flies in the adjacent field, illustrating the potential value of *E. muscae* for control strategies that build on the enhancement of natural enemies in non-crop reservoirs (Eilenberg and Philipsen 1988; Pell et al. 2010). Hedges and other vegetation adjacent to crops are also known as important refuges for SWD (Baroffio et al. 2014; Diepenbrock and Burrack 2017; Kenis et al. 2016), and thus zones where flies may be concealed from conventional pest control strategies used in the field and particularly could get attacked by pathogens and other natural enemies. Moreover, as SWD uses bushes and woods as overwintering sites (Pelton et al. 2016; Briem et al. 2016), a decrease in flies through the presence of pathogens might delay the build-up of dense populations early in season.

As *E. muscae* is able to infect and kill SWD and furthermore is known to cause epizootics in other dipteran species, it is relevant to contemplate if members of the *E. muscae* species complex could be used in biological control. Attributes that generally are considered as beneficial for the application of entomophthoralean fungi are a specialized host range, the potential to cause epizootics and the existence of persisting resting spores (Hajek and Delalibera 2010). However, many Entomophthorales are difficult to mass-produce and grow in vitro (Hajek et al. 2012), and so far devices for autoinoculation have been developed against dipteran pests including SWD only with hypocrealean entomopathogenic fungi (Maniania et al. 2006; Migiro et al. 2010; Yousef et al. 2017). Therefore, control measures operating by release of infected animals that disseminate the pathogens, or enhancement of entomophthoralean fungi in the environment of agroecosystems (e.g. by providing refuges adjacent to crops) might be the most practical way to circumvent the challenging development of formulations for spray application (Tobin and Hajek 2012; Zúbrik et al. 2016). An advantageous attribute for application in pest control is that members of the *E. muscae* species complex are dipteran-specific, which in comparison with generalist pathogens implies a smaller range of susceptible non-target species. Intricate molecular interactions underlying host-specific adaptation of *E. muscae* have led to a more narrow host range as compared to generalist hypocrealean fungi such as *M. robertsii* and *B. bassiana* (De Fine Licht et al. 2017; Hansen and De Fine Licht 2017).

Feasible biological control with the here tested isolate of *E. muscae* would require a pathogen host shift from the indigenous host species, *Musca domestica,* on to the invasive SWD. Should a host shift as generated in the laboratory also occur in the field it would be highly beneficial and potentially provide the basis for further development of biological control measures. Other isolates within the *E. muscae* species complex are known to naturally infect Drosophilid species (Goldstein 1927; Turian and Wüest 1969). Naturally infected species of the genus *Drosophila* have rarely been collected, but would provide an ideal starting point for developing new diptera-specific biological control strategies for SWD.

## Author contribution statement

PGB and HHDFL designed the study and wrote the manuscript. REJ, MEN and VV performed the assays. REJ and MEN analysed the conidia exposure and morphology data, VV analysed the survival and mortality data. All authors contributed to the writing of the final version of the manuscript.
